# Differential Behavior of Non-*albicans Candida* Species in the Central Nervous System of Immunocompetent and Immunosuppressed Mice

**DOI:** 10.3389/fmicb.2018.02968

**Published:** 2019-01-08

**Authors:** Marcelo D’Alessandre Sanches, Luiza A. N. Mimura, Larissa R. C. Oliveira, Larissa L. W. Ishikawa, Hans G. Garces, Eduardo Bagagli, Alexandrina Sartori, Cilmery Suemi Kurokawa, Thais F. C. Fraga-Silva

**Affiliations:** ^1^Botucatu Medical School, São Paulo State University (UNESP), Botucatu, Brazil; ^2^Institute of Biosciences, São Paulo State University (UNESP), Botucatu, Brazil

**Keywords:** *Candida* spp., fungal infections, prednisolone, neuroinflammation, microglia

## Abstract

The genus *Candida* includes commensal fungi that can cause local and systemic infections, frequently involving vital organs as the central nervous system (CNS). *Candida* spp. occupy the fourth place among infections that affect the CNS. Although the incidence of *Candida albicans* is decreasing among patients under immunosuppressive therapies, the incidence of non-*albicans Candida* is increasing. In this context, the objective of this work was to evaluate the ability of non-*albicans Candida* species to spread to the CNS of immunocompetent and immunosuppressed mice. Adult female C57BL/6 mice were treated with prednisolone, intravenously infected with *Candida glabrata*, *Candida krusei* and *Candida parapsilosis* yeasts and then evaluated at the 3rd and 14th days after infection. All *Candida* species disseminated to the brain from immunocompetent animals and induced local inflammation at the third day post-infection. The immunosuppression resulted in body weight loss, leukopenia and reduced IL-2 production by spleen cell cultures. Higher fungal loads were recovered from the CNS of immunosuppressed mice. Inflammatory infiltration associated to a Th1 subset profile was higher in brain samples from *C. krusei* immunosuppressed mice compared with immunocompetent ones. Additionally, *C. krusei* was able to transform into pseudohypha inside microglia *in vitro* infected cells and also to induce elevated nitric oxide production. Altogether, these results indicate that *C. glabrata*, *C. krusei* and *C. parapsilosis* are able to disseminate to the CNS and promote local inflammation in both immunocompetent and immunosuppressed mice. *C. krusei* displayed a distinct behavior at the CNS triggering a local Th1 profile. The possible contribution of these non-*albicans Candida* species to other CNS pathologies as multiple sclerosis, Parkinson’s and Alzheimer’s diseases deserves further attention.

## Introduction

The genus *Candida* includes commensal fungi that live in the human oral cavity, gastrointestinal and genitourinary tracts. In certain conditions, such as changes in the microbiota and loss of integrity of epithelial barriers, their excessive growth or translocation through the intestine may occur and cause local and systemic infections (Eggimann et al., 2003). Thus, *Candida* spp. are considered opportunistic commensal pathogens that promote infection under host predisposing conditions.

Systemic *Candida* infections affect vital organs, including the central nervous system (CNS) (Li et al., 2017). Morbidities in these infections are severe and comprise a broad spectrum of clinical symptoms, including brain abscesses, meningitis/meningoencephalitis, stroke/vasculitis, and death (Murthy and Sundaram, 2014). The spread of this pathogen to the brain is more common in newborns (Faix and Chapman, 2003) and this is also responsible for most of the brain abscesses in immunocompromised patients (Yampolsky et al., 2010). *Candida* spp. occupy the fourth place among infections in the CNS (Panackal and Williamson, 2015). Non-*albicans Candida* species are also found in the CNS and the most frequent pathogenic species are *Candida glabrata*, *Candida parapsilosis*, *Candida tropicalis* and *Candida krusei* (Storti et al., 2012; Pfaller et al., 2014; Turner and Butler, 2014). The most common risk factors for these infections are associated with the use of catheters (venous, urinary and arterial), administration of broad-spectrum antibiotics, mechanical ventilation, nasogastric intubation, enteral and parenteral nutrition, and treatment with systemic corticosteroids (Jordán et al., 2014).

To invade the CNS, fungi must cross the blood-brain barrier (BBB). *Candida albicans* is able to pass through the human brain microvascular endothelial cells (HBMECs) via transcytosis and it is also able to generate pseudohyphae inside these cells (Jong et al., 2001). This process requires the initial binding of fungal invasins, ALS3 and SSA1, to proteins expressed in HBMECs. ALS3 binds to the gp96 heat shock protein whereas SSA1 binds to other receptor in HBMECs (Liu et al., 2011). These proteins were initially described and studied only in *C. albicans*. ALS3 was later also identified in non-*albicans Candida* species, including *C. dubliniensis* and *C. tropicalis* (Hoyer et al., 2001) and an ALS3 homolog (CpALS7) was described in *C. parapsilosis* (Bertini et al., 2016).

Neurodegenerative pathologies such as Alzheimer’s disease, Parkinson’s disease, amyotrophic lateral sclerosis and multiple sclerosis (MS), constitute a heterogeneous group of CNS disorders that are characterized by a slow and irreversible loss of neuronal functions. Although their etiology remains largely unknown (Pisa et al., 2015), some studies suggested that CNS infections could act as cofactors in the development of these diseases (Mattson, 2012; Saroukolaei et al., 2016; Góralska et al., 2018). Evidences observed in MS patients support the possible contribution of *Candida* infections to the development of neurodegenerative and/or autoimmune diseases. Compared to healthy subjects, MS patients present significantly higher levels of specific antibodies and *Candida* spp.-derived macromolecules in blood (Benito-León et al., 2010; Pisa et al., 2011) and in the cerebrospinal fluid (Pisa et al., 2013).

On the other hand *Candida* spp. infections in MS patients could be a consequence of disease treatment since therapy is mostly relied on immunosuppression to reduce the frequency and severity of exacerbations. Thus, MS acute episodes are usually treated with corticosteroids whereas chronic periods are controlled by immunomodulatory drugs (Gold and Wolinsky, 2010). In this context, prednisolone is a glucocorticoid employed to treat a plethora of chronic inflammatory diseases (De Kruif et al., 2007; Yan et al., 2015) whose anti-inflammatory role is due to its ability to suppress or inhibit the presence of NF-kB transcription factors (Andersson et al., 2005). This possible relationship between immunosuppression triggered by prednisolone therapy with increased fungal load and also with systemic dissemination was recently described in a murine model of gastrointestinal candidiasis (Kobayashi-Sakamoto et al., 2018). In this scenario we could think that fungal infections, including the ones triggered by non-*albicans Candida* species, could be involved in disease development by previous infections or disease exacerbation by infections associated with immunosuppressive therapy.

Interestingly, the incidence of *C. albicans* decreased in many countries, especially among patients under immunosuppressive therapies, while the incidence of non-*albicans Candida* clearly increased (Yapar, 2014; Wu et al., 2017). Similarly to *C. albicans*, non-*albicans Candida* species can also reach the CNS, opening up the interesting possibility that they can present distinct neurotropism and also a specific behavior considering their spread to the CNS when hosts are under immunosuppressive therapy. Therefore, in the present study we evaluated the ability of three non-*albicans Candida* species to spread to the CNS in immunocompetent and immunosuppressed C57BL/6 mice. As expected, immunosuppression enhanced fungal burden in the CNS but *C. glabrata*, *C. krusei* and *C. parapsilosis* species behaved differently.

## Materials and Methods

### Animals

Female C57BL/6 mice with 9 to 11 weeks old were purchased from a specific pathogen-free facility at University of São Paulo (USP) (Ribeirão Preto, SP, Brazil). Mice were allocated in cages (maximum five animals per cage) with sterilized food and water *ad libitum* and were manipulated in accordance with the local Ethics Committee on Use of Animals (CEUA), from São Paulo State University (UNESP) (Botucatu, SP, Brazil).

### Experimental Design

For initial evaluations, 40 immunocompetent mice were allocated into eight groups, with 5 mice per group: 3 days non-infected and 3 days infected mice with *C. glabrata*, *C. krusei* and *C. parapsilosis* and 14 days non-infected and 14 days infected mice with *C. glabrata*, *C. krusei* and *C. parapsilosis*. In the subsequent analyses were used 32 immunocompetent (control - CTL) and 32 immunosuppressed (prednisolone – PRED) animals. Mice were allocated into 4 CTL and 4 PRED groups (8 mice per group): non-infected mice, mice infected with *C. glabrata*, mice infected with *C. krusei* and mice infected with *C. parapsilosis*. Clinical parameters were followed for 14–16 days. Immunological and histopathological evaluations were performed at 14 days after infection and 11 days after prednisolone treatment.

### *Candida* spp. Inoculum

*Candida glabrata* (H-3479), *C. krusei* (ATCC 6258), and *C. parapsilosis* (ATCC 90018) were obtained from the fungal collection of Mycology Laboratory (UNESP, Botucatu, SP, Brazil) where they were stored at -80°C in glycerol stocks. For mice infection, *Candida* spp. were cultured in Sabouraud-dextrose agar (Difco Laboratories, Detroit, MI, United States) for 24 h, at 37°C, and carefully washed with sterile saline solution (SSS). The suspensions were centrifuged and washed twice at 450 ×*g* for 5 min at 4°C. The fungal concentration was adjusted to 5.0 × 10^7^ viable yeast cells/mL in SSS and then inoculated into the lateral tail vein (0.1 mL/animal). Fungal suspensions were previously heat-killed to be used for culture stimulation.

### Immunosuppression

The animals were immunosuppressed by administration of two subcutaneous injections of prednisolone (Sigma-Aldrich, St. Louis, MO, United States) at a dose of 100 mg/kg body weight delivered 1 day before and 3 days after the infection with *Candida* spp., according to Takakura et al. (2003).

### Determination of Fungal Burden

Fourteen days after infection, mice were anesthetized with ketamine/xylazine and perfused with 10 mL of SSS. Brain, spinal cord and spleen were collected, weighed and macerated in 1.0 mL of SSS. Afterward, 0.1 mL from each tissue homogenate was spread over culture plates containing Sabouraud-dextrose agar (Difco) using a Drigalski T loop. The plates were then sealed and incubated at 37°C for 3 days. The number of colony forming units (CFU) was normalized per gram of tissue and log transformed.

### Spleen Cell Culture

Spleens were initially dissociated with sterile pestles and then the red blood cells were lysed with buffer containing NH_4_Cl. The remaining cells were washed with RPMI 1640 (Sigma-Aldrich) medium and the cells were adjusted to 5 × 10^6^ cells/mL in RPMI medium supplemented with 10% heat-inactivated fetal calf serum (Gibco BRL, Grand Island, NY, United States), 2 mM of L-glutamine (Sigma-Aldrich) and 0.1% antibiotic-antimycotic (Sigma-Aldrich). Spleen cell cultures were stimulated with heat-killed *Candida* spp. (5 yeasts/cell) and incubated at 37°C, 5% CO_2_ in a humidified incubator for 48 h. Cell-free supernatants were harvested and stored at -20°C for cytokine analysis.

### Cytokine Quantification

Cytokine levels were evaluated by enzyme-linked immunosorbent assay (ELISA) in culture supernatants using IFN-γ, IL-5 and IL-10 BD OptEIA Sets (Becton, Dickinson and Company, BD, Franklin, San Diego, CA, United States) and IL-2, IL-6, IL-17, and TNF-α Duosets (R and D Systems, Minneapolis, MN, United States). The assays were performed according to the manufacturer’s instructions.

### Histopathology of the CNS

After euthanasia, brain and lumbar spinal cord samples were removed and fixed in 10% neutral buffered formalin. Paraffin slides with 4 μm were stained with hematoxylin and eosin (HE) to evaluate the inflammatory process. A semi-quantitative analysis of CNS inflammation was performed according to the following criteria: (0) inflammatory infiltration absent; (1) mild inflammatory infiltration; (2) moderate inflammatory infiltration, and (3) intense inflammatory infiltration.

### RT-qPCR

Frozen brain samples were used for RNA extraction with Trizol reagent (Life Technologies, Carlsbad, CA, United States) and cDNA synthesis (RT-PCR) was performed according to the manufacturer’s recommendations (High Capacity RNA-to-cDNA converter kit, Life Technologies). Expression of *Gata3*, *Rorc*, *Foxp3*, *Tbx21*, *IFN-y*, *TNF-α*, *CLEC7A (dectin-1)*, *TLR-2*, *TLR-4* genes was analyzed based on GAPDH reference gene levels. Real Time PCR was performed using Taqman system according to manufacturer’s recommendations (Applied Biosystems, Foster City, CA, United States). Gene expression was represented as relative fold difference (2^-ΔΔCt^) using the Control (non-treated and non-infected) group as the calibrator.

### Biofilm Quantification by the XTT Assay

*Candida albicans* (SC5314) and the three non-*albicans Candida* species were grown in YEPD broth (Difco, Sparks, MD, United States) during 24 h. The inoculum was standardized at a concentration of 10^7^ cells/mL in RPMI 1640 (Sigma-Aldrich) medium. The specimens (0.1 mL of inoculum) were plated in triplicate in 96-well cell culture and incubated at 37°C, 5% CO_2_ in a humidified incubator for 24h. After incubation time intervals of 6, 18, and 24 h, the specimens were carefully washed three times with 0.1 mL of phosphate-buffered saline (PBS) and the biofilm formed was evaluated by the metabolic activity according to the reduction of XTT to formazan. XTT solution, 0.1 mL at 0.5 mg/mL plus menadione 0.1 mM (Sigma-Aldrich^®^ Inc.), were added to each well and the plates were covered with aluminum foil and incubated at 37°C for 3 h. Then, 200 μl were plated in triplicate in a 96-well plate and analyzed by absorbance at 550 nm using a chemiluminescence microreader (ELx 800; BioTek Instruments Inc., Winooski, VE, United States).

### Microglial Cell Culture and NO Quantification

The BV-2 cell line (BCRJ code 0356) was used to evaluate nitric oxide (NO) induction by *Candida* spp. *in vitro*. *C. albicans* (SC5314) and the three non-*albicans Candida* species were grown in YEPD broth (Difco) during 24 h. BV-2 cells were plated on glass slides into 24-well plates at 5 × 10^5^ cells/well in DMEM High Glucose medium supplemented with 10% heat-inactivated fetal calf serum (Gibco BRL) and 1% of sodium pyruvate (Sigma-Aldrich). After 2.5 h, *Candida* spp. were added in the same proportion, 5 × 10^5^ yeast/well, and the plate was incubated in 5% CO_2_ in a humidified incubator. After incubation time intervals of 6, 18 or 24 h, the supernatants were collected and NO levels were determined according to the method described by Green et al. (1981) (41) and cells adhered in the glass slides were stained with panoptic (Laborclin, Pinhais, PR, Brazil). Nitrite accumulation was then quantified using a chemiluminescence microreader (ELx 800; BioTek Instruments Inc., Winooski, VE, United States). The concentration of nitrite was determined using sodium nitrite (Sigma-Aldrich) diluted in distilled water as a standard.

### Phylogenetic Analysis

**FIGURE 1:**
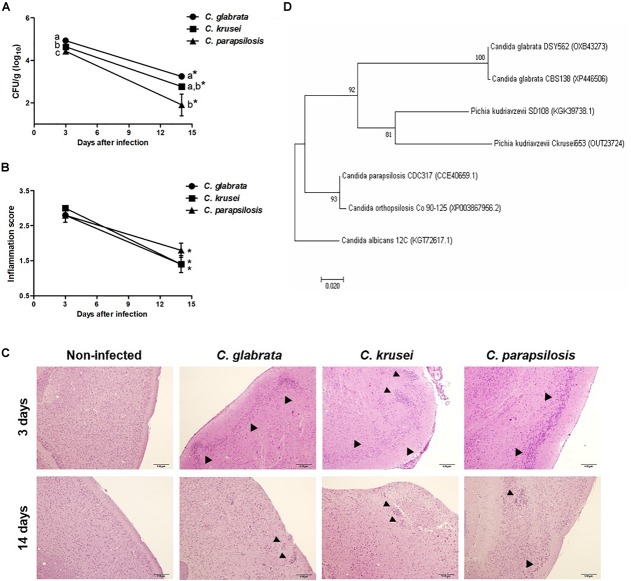
Non-*albicans Candida* dissemination to the brain in immunocompetent mice. Brain fungal load **(A)**, inflammation score **(B),** and inflammatory infiltration **(C)** of *Candida glabrata*, *Candida krusei*, and *Candida parapsilosis* in brain samples were evaluated at 3 and 14 days after infection. The results are expressed as mean ± SEM; ^∗^indicate statistical difference between 3 and 14 days analyzed by unpaired *t*-test; different letters indicate statistical difference among groups analyzed by One-way ANOVA with *post hoc* Tukey test; *p* < 0.05; *n* = 5/group. Arrowheads indicate diffuse inflammatory infiltration and focus of inflammatory infiltration. Phylogenetic construction of SSA1 protein **(D)** for *C. glabrata*, *C. krusei* (*Pichia kudriavzevii*) and *C. parapsilosis*. The percentage of trees in which the associated taxa clustered together is shown next to the branches.

Sequences deposited at the Protein Database of the NCBI (National Center for Biotechnology and Information) were obtained for analyzed species by blasting at the BLAST tool^1^ of the same institution by using the protein sequence of SSA1 protein of *Saccharomyces cerevisiae* S288C (accession number NP009396.2). The sequences were aligned with a PAM matrix and the phylogenetic construction was made by using the Maximum Likelihood method based on the Le and Gascuel model (Le and Gascuel, 2008) applying a bootstrap of 1000 replicates (Felsenstein, 1985). *Candida albicans* 12C (Accession number KGT72617.1) was used as outgroup. The accession number of each sequence is included within parenthesis in Figure 1D. The analysis was made by using the software MEGA v7.0 (Kumar et al., 2016).

### Statistical Analysis

The results were initially analyzed by Shapiro-Wilk’s test, to test for the normality of data. Comparisons between two samples were made by *t*-test and three or more samples were made by One Way ANOVA followed by Tukey’s test. Data were analyzed using SigmaPlot statistical package for Windows version 12.0 (Jandel Corporation, CA, United States) and values of *p* < 0.05 were considered statistically significant. Graphs and figures were made in GraphPad Prism 7 (GraphPad Software Inc., San Diego, California, United States). Results were expressed as mean ± standard error of mean (SEM).

## Results

### Non-*albicans Candida* Disseminate to the Brain of Immunocompetent Mice

Mice were infected with *C. glabrata*, *C. krusei* or *C. parapsilosis* and evaluated 3 and 14 days after infection. All three *Candida* spp. were able to spread to the brain and to trigger a local inflammatory process at the third day. As shown in Figure 1A, the three species presented a significantly decreased fungal load on day 14. *C. glabrata* fungal load was the highest, *C. parapsilosis* was the lowest and *C. krusei* presented an intermediate burden in both analyzed periods. Histopathologic scoring revealed that inflammation, similarly to fungal load, was also decreased 14 days after infection (Figure 1B). As illustrated at Figure 1C, all three *Candida* spp. induced inflammation at the brain meninges and parenchyma (gray matter and white matter). As SSA1 is a fungal invasin that allows *C. albicans* trafficking to the brain, we used a bioinformatics approach to investigate the presence of this protein in the three non-*albicans Candida* species. The phylogenetic construction performed considering the SSA1 protein revealed that *C. parapsilosis* is phylogenetically more distant comparing to the other two species (*C. glabrata* and *C. krusei*) that clustered together within the same clade (Figure 1D).

### Prednisolone Affects Body Weight, Leukocyte Number and IL-2 Production

**FIGURE 2:**
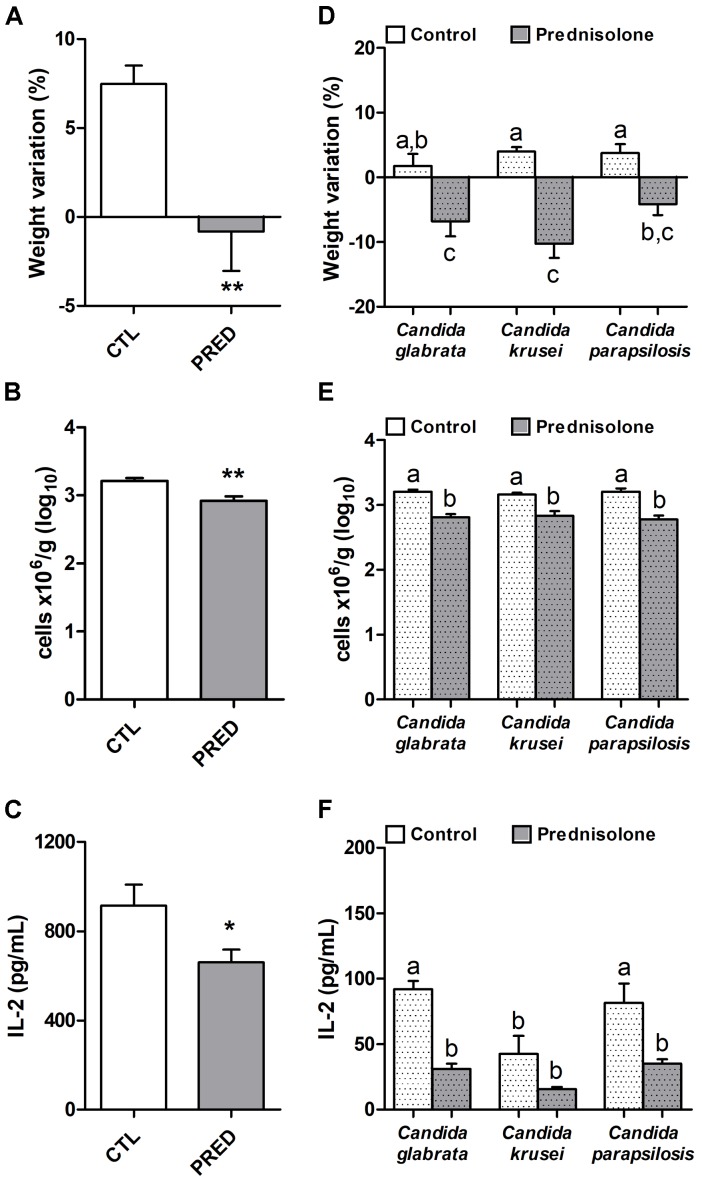
Effect of prednisolone in non-infected and infected mice. Body weight **(A,D)**, total spleen leukocyte cell number **(B,E)** and production of IL-2 by spleen cell cultures **(C,F)** in immunocompetent (CTL – control) and immunosuppressed (PRED – Prednisolone) non-infected and infected mice. The results are expressed as mean ± SEM; ^∗^*p* < 0.05 and ^∗∗^*p* < 0.01 statistical difference between groups analyzed by *t*-test; different letters indicate statistical difference among groups analyzed by One-way ANOVA with *post hoc* Tukey test; *p* < 0.05; *n* = 8/group.

Prednisolone treatment was associated with significant body weight loss in normal (non-infected) and also in mice infected with any of the three *Candida* species, as shown in Figures 2A and D, respectively. Efficacy of corticotherapy was supported by reduction in both, splenic leukocyte number and IL-2 production. Lower cell numbers are illustrated at Figures 2B and E for normal and infected mice, respectively. Decreased IL-2 production is displayed in Figures 2C and F for normal and infected mice, respectively.

### Non-*albicans Candida* Species: Biofilm Formation and Prednisolone Effect on Fungal Load and Cytokine Production in Spleen

**FIGURE 3:**
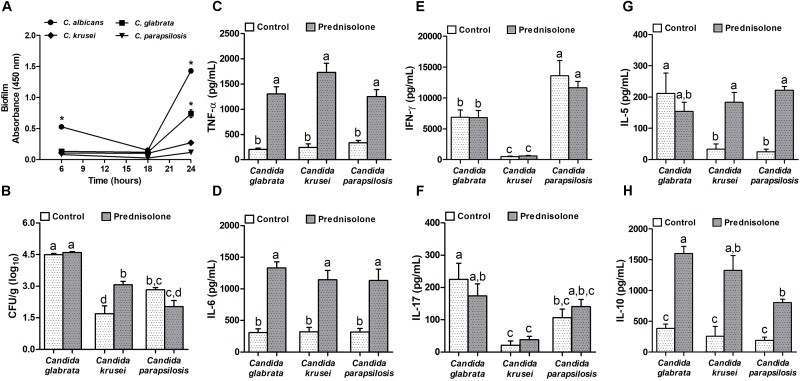
Non-*albicans Candida* species: biofilm formation and effect of prednisolone on fungal load and cytokine production in spleen. Absorbance of *in vitro* biofilm assay **(A)**, spleen fungal load **(B)**, and production of TNF-α **(C)**, IL-6 **(D)**, IFN-γ **(E)**, IL-17 **(F)**, IL-5 **(G)** and IL-10 **(H)** by spleen cell cultures of immunocompetent (Control) and immunosuppressed (Prednisolone) infected-mice. The results are expressed as mean ± SEM; ^∗^ and different letters indicate statistical difference among groups analyzed by One-way ANOVA with *post hoc* Tukey test; *p* < 0.05; *n* = 8/group or *n* = 3/group in biofilm assay.

Concerning biofilm formation, *C. albicans* was introduced in this experiment as a positive control. The biofilm formation was checked at different time points. As expected, *C. albicans* was already able to form biofilm after 6 h when biofilm was not detected in non-*albicans Candida* species. After 24 h incubation, a very clear difference was observed and indicated that, similarly to *C. albicans*, *C. glabrata* was also able to trigger biofilm formation (Figure 3A). Distinctly from these species, *C. krusei* and *C. parapsilosis* had a poor biofilm forming ability (Figure 3A). This elevated ability to trigger a biofilm coincided with the highest splenic fungal load determined by *C. glabrata* (Figure 3B). Differently from *C. glabrata* and *C. parapsilosis* whose splenic fungal burdens were not affected by the corticoid treatment, *C. krusei* fungal load was significantly higher in prednisolone-treated mice (Figure 3B). From the six tested cytokines, four of them, TNF-α, IL-6, IL-5 and IL-10, were significantly higher in prednisolone-treated mice (Figures 3C,D,G,H); with exception of IL-5 in *C. glabrata* infected mice. Differently from other cytokines, IFN-γ and IL-17 production was not affected by corticotherapy (Figures 3E,F). However, IFN-γ and IL-17 levels were clearly different in *C. krusei* infection; these cytokines were significantly lower in *C. krusei* in both, normal and immunosuppressed mice, in comparison to *C. glabrata* and *C. parapsilosis*.

### Prednisolone Therapy Enhances Fungal Load and Inflammation at the CNS

**FIGURE 4:**
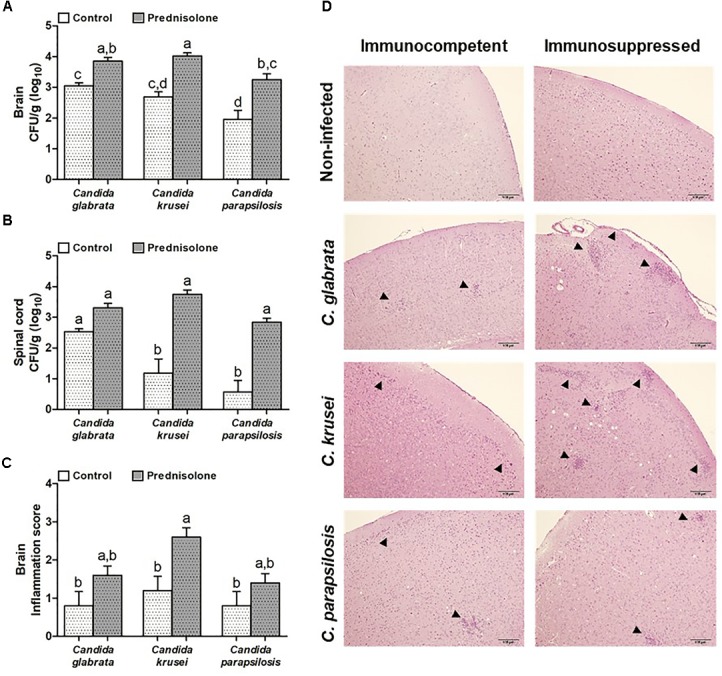
Effect of prednisolone on fungal load and inflammation at the CNS. Fungal load of *C. glabrata*, *C. krusei* and *C. parapsilosis* in brain **(A)** and spinal cord **(B)** samples and inflammation score **(C)** in brain **(D)** of immunocompetent and immunosuppressed infected mice. The results are expressed as mean ± SEM (different letters indicate statistical difference among groups analyzed by One-way ANOVA with *post hoc* Tukey test; *p* < 0.05; *n* = 8 for CFU and *n* = 5 for inflammation score). Arrowheads indicate diffuse inflammatory infiltration and focus of inflammatory infiltration.

Mice infected with *C. glabrata*, *C. krusei* or *C. parapsilosis* were treated with prednisolone and evaluated 14 days after the infection. Prednisolone treatment usually increased fungal load at both, the brain (Figure 4A) and the spinal cord (Figure 4B). The only exception was the amount of fungi recovered from the spinal cord of *C. glabrata*-infected mice that was similar in immunocompetent and immunosuppressed mice (Figure 4B). Treatment with prednisolone significantly increased brain inflammation only in *C. krusei* infected mice (Figure 4C). Presence of inflammatory infiltrates in the brain of immunocompetent and immunosuppressed mice is represented in Figure 4D. Spinal cord from infected mice presented no visible inflammation when analyzed in HE stained sections.

### *C. krusei* Infection Triggers Th1 Neuroinflammation in Immunosuppressed Mice

**FIGURE 5:**
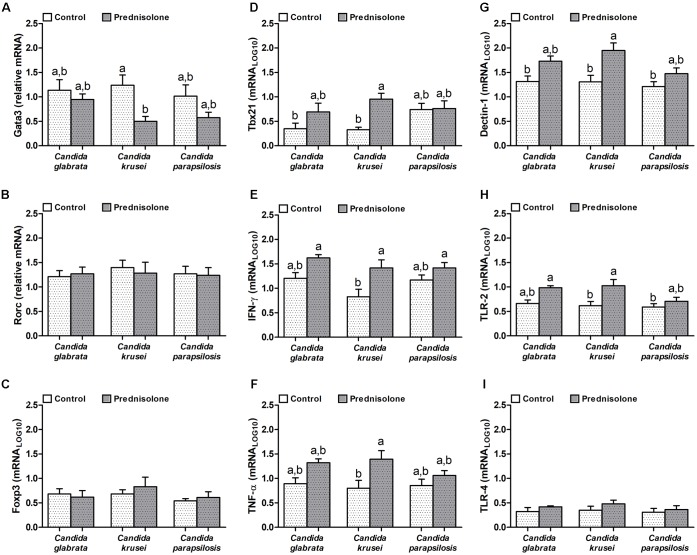
Increased mRNA expression for pro-inflammatory markers in the brain of immunosuppressed *C. krusei* infected mice. Relative mRNA expression of GATA3 **(A)**, RORc **(B)**, Foxp3 **(C)**, Tbx21 **(D)**, IFN-γ **(E)**, TNF-α **(F)**, Dectin-1 **(G)**, TLR-2 **(H)**, and TLR-4 **(I)** in brain samples of immunocompetent (Control) and immunosuppressed (Prednisolone) infected mice. The results are expressed as mean ± SEM (different letters indicate statistical difference among groups analyzed by One-way ANOVA with *post hoc* Tukey test; *p* < 0.05; *n* = 6).

Quantification of mRNA transcription factors indicated that Gata3 (Th2 signature) was downmodulated by prednisolone in *C. krusei*-infected mice (Figure 5A). Rorc (Th17 signature) and Foxp3 (Treg signature) were similarly expressed in the brain of prednisolone treated and non-treated infected mice (Figures 5B,C). In addition, no differences were observed concerning the three *Candida* spp. However, clear differences were detected when the Th1 subset was evaluated. *C. krusei* immunosuppressed mice showed a higher mRNA expression of Tbx21, IFN-γ and TNF-α compared to *C. krusei* immunocompetent mice (Figures 5D–F). Additionally, this group showed a higher mRNA expression of Dectin-1 and TLR-2 compared to *C. krusei* immunocompetent mice (Figures 5G,H), but no difference was observed in TLR-4 mRNA expression (Figure 5I).

### *C. krusei* Induces Higher NO Production and Yeast-to-Hyphae Transition in Microglia

**FIGURE 6:**
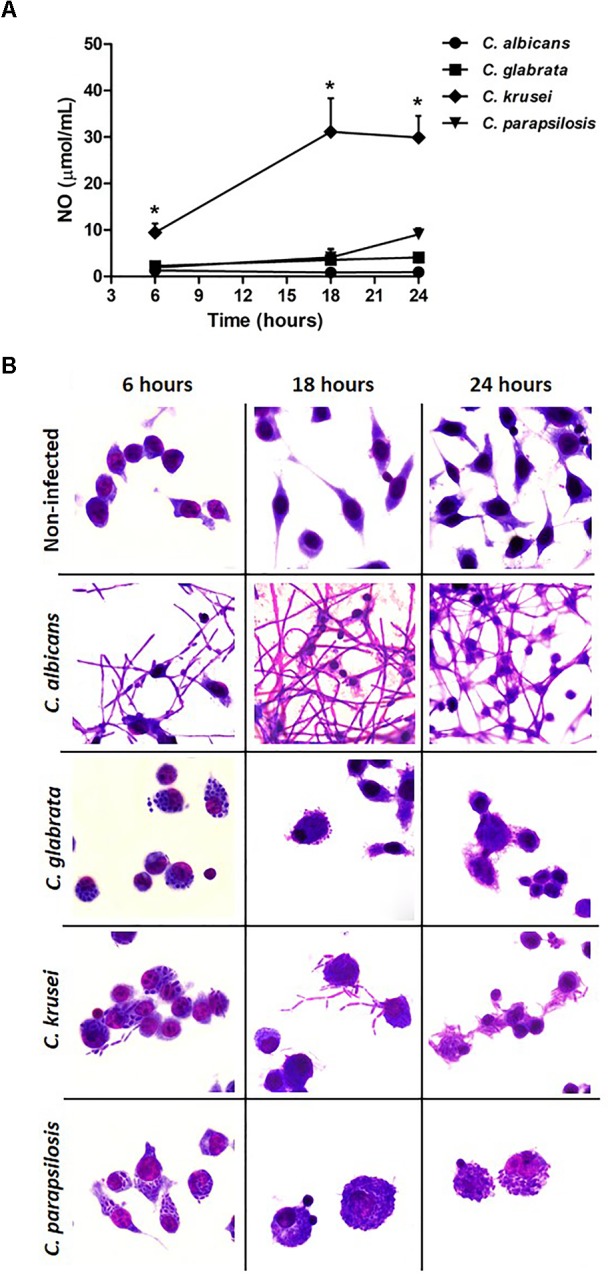
*Candida krusei* triggers higher NO production and yeast-to-hyphae transition in microglia. Nitric oxide (NO) levels **(A)** were measured in the supernatant of microglial cell (5 × 10^5^/well) co-cultured with *C. albicans*, *C. glabrata*, *C. krusei* and *C. parapsilosis* (5 × 10^5^ yeasts/well) at 6, 18, and 24 h after incubation **(B)**. The results are expressed as mean ± SEM (^∗^ indicate statistical difference among groups analyzed by One-way ANOVA with *post hoc* Tukey test; *p* < 0.05; *n* = 3/group).

A microglial cell lineage (BV-2) was infected with the different *Candida* species, including *C. albicans*, to test and compare their ability to produce nitric oxide (NO) and also their potential to go through yeast-to-hypha transition. *C. krusei* was clearly the most potent NO inducer; its levels were clearly different from the other species being significantly elevated after 6, 18, and 24 h after infection (Figure 6A). *C. albicans* was used as a control and, as expected, hypha formation was already occurring after 6 h of incubation. At this time-point, a transition from yeast-to-hypha was already beginning at *C. krusei* infected cells. As indicated by micrographs, pseudohypha of *C. krusei* are already coming out of the BV-2 cells after 18 and 24 h. The yeast-to-hypha transition was not detected in *C. glabrata* and *C. parapsilosis* infected cells in any tested time-points. These results are illustrated at Figure 6B.

## Discussion

The prevalence of fungal infections is increasing during the past few years. Even though some fungi can trigger pathologies in health subjects, most of these agents provoke illness in previously immunocompromised hosts (Góralska et al., 2018). High risk groups include HIV-infected persons and aids patients, transplant receivers and patients under immunosuppression therapy (Lionakis and Kontoyiannis, 2003; Chastain et al., 2017; Nel et al., 2018). The involvement of the CNS during fungal infections is primarily found in immunocompromised hosts being *Cryptococcus*, *Aspergillus*, and *Candida* more frequently identified (Henao and Vagner, 2011; Gavito-Higuera et al., 2016).

In a previous report we demonstrated that a systemic infection by *Candida albicans* disseminates to the CNS and significantly increases the severity of experimental autoimmune encephalomyelitis that is a mouse model for MS (Fraga-Silva et al., 2015). As the potential of non-*albicans Candida* species to reach the CNS is not being properly investigated, we used experimental murine infection to compare the ability of *C. glabrata*, *C. krusei* and *C. parapsilosis* to reach the CNS and to cause a local inflammation. To mimic the more frequent situation in which installation at the CNS is more prevalent in immunosuppressed subjects, mice were also acutely immunosuppressed by prednisolone therapy just before experimental infection.

All three non-*albicans Candida* species were able to reach the brain in immunocompetent mice and to cause local inflammation. However, both fungal and inflammation clearly subsided 2 weeks after infection. As *C. glabrata* was present in the brain in higher amount than the two other species, their ability to form a biofilm was compared *in vitro*. The biofilm forming ability of *C. glabrata* was elevated being similar to the one formed by *C. albicans*, which was used as a positive control, and could, at least partially, explain its higher fungal load at both brain and spleen. This finding is important because after *C. albicans*, *C. glabrata* is considered one of the most prevalent pathogenic fungi in the human population (Rodrigues et al., 2017). In addition, its strong capacity to form biofilm is described as one of its most consistent virulence factors (Fonseca et al., 2014). The SSA1 protein, an invasin-like molecule, plays a central role in the virulence of *Candida*. This protein is important for brain invasion since it also induces endocytosis by HBEMCs (Liu et al., 2011). A bioinformatics approach allowed us to identify this protein in the three non-*albicans Candida* species and also to build a phylogenetic tree. This construction revealed that *C. parapsilosis* is phylogenetically more distantly related to the other two species (*C. glabrata* and *C. krusei*) that clustered together within the same clade. These results are in agreement with the fungal loads in the brain since the three species were able to reach the CNS but the fungal burdens differed among them.

To investigate the effect of an acute state of immunosuppression on peripheral fungal load and immune response, we employed the prednisolone treatment protocol described by Takakura et al. (2003). This procedure significantly reduced body weight in both, normal and infected mice with any of the three *Candida* species. This finding indicated that corticotherapy was efficient since it is well documented that this drug triggers catabolic effects on skeletal muscles (Sali et al., 2012). A reduced splenic cell number in both, infected and non-infected mice, additionally supported the effectiveness of corticotherapy. Even though the total number of T cells in the spleen was not determined, the reduced spleen cell number was possibly due to T cell apoptosis that has been consistently associated with corticosteroid effects (Herold et al., 2006). This possibility was reinforced by the decreased production of IL-2, that is the main T cell growth factor and that is also produced by these cells. Inhibition of cytokine production, including IL-2 production, is broadly accepted as a very relevant mechanism involved in corticoid immunosuppressive effect (Riccardi et al., 2002).

The analysis of peripheral cytokine production together with the splenic fungal load suggested that these three non-*albicans Candida* species interact distinctly with the host. Considering IFN-γ production, for example, that is required for optimal phagocyte activation and favors the induction of Th1 protective response in *C. albicans* infections (Gozalbo et al., 2014), the results indicated that *C. glabrata* and *C. parapsilosis* induce significant levels of this cytokine. Interestingly, *C. parapsilosis* induces more IFN-γ and controls better the infection. Contrasting with the two other tested species, *C. krusei* induced very low levels of IFN-γ and IL-17 that are signature cytokines denoting Th1 and Th17 presence, respectively (Raphael et al., 2015), suggesting that, at least at the periphery, this infection is not controlled by the classical Th1 and Th17 subsets.

Unexpectedly, an elevated production of some cytokines was observed precisely at the groups that were treated with prednisolone. This was the case of TNF-α, IL-6 and IL-10 in immunosuppressed mice infected with the three species and of IL-5 in immunosuppressed mice infected with *C. krusei* and *C. parapsilosis*. A possible differential effect of prednisolone in innate and adaptive immunity could explain this finding. We therefore suggest that the very well described and established downmodulatory effect of corticosteroids on adaptive immunity (Coutinho and Chapman, 2011; Olnes et al., 2016) is associated with a concomitant enhancement of, at least, some aspects of innate immunity. High doses of glucocorticoids induced apoptosis of T cells and other immune cells such as thymocytes, B cells, macrophages, mature dendritic cells, eosinophils, and natural killer cells (Gruver-Yates and Cidlowski, 2013), but did not affect monocytes and neutrophils (Serra-Bonett et al., 2009). Even though the literature concerning this subject is very scarce and also indirect, this possibility is supported by some findings as the presence of neutrophilic leukocytosis in children with nephrotic syndrome being treated with systemic corticosteroids (Bariş et al., 2016) and enhanced survival and function of neutrophils and alveolar macrophages by inhaled glucocorticoids (Schleimer, 2004). Interestingly, during systemic candidiasis there is an accumulation of neutrophils in all organs, except the brain (Lionakis et al., 2011) and these cells are able to produce a plethora of cytokines including, for example, TNF-α, IL-6, IL-10 and IL-5 (Tecchio et al., 2014; Xu et al., 2017) that were found elevated in spleen cell cultures from prednisolone treated mice, as described above.

Contrasting with the effect of corticotherapy on spleen fungal recovery that increased only the amount of *C. krusei*, the prednisolone treatment determined a significant increase in the CNS fungal load of the three *Candida* species. This findings seems very relevant because it strongly supports that corticotherapy, and possible other immunosuppressive drugs, are able to facilitate fungal dissemination to the CNS as has already being evidenced (Sharma, 2010). CNS invasion by these non-*albicans Candida* species deserves attention and investigations concerning not only their contribution as causes of meningitis, cerebritis and abscess formation (Gavito-Higuera et al., 2016) but also as possible triggers or aggravators of neurodegenerative pathologies as Alzheimer (Fulop et al., 2018) and MS (Benito-León and Laurence, 2017).

In spite of this similar and significant dissemination of these *Candida* species to the CNS in immunosuppressed mice, a very distinct local inflammatory scenario was observed in the brain. *C. glabrata* and *C. parapsilosis* behaved similarly, that is, they triggered a slightly increased inflammation in previously suppressed mice. However, the brain of mice treated with prednisolone before *C. krusei* infection displayed a significantly enhanced inflammatory infiltrate. The local analysis of mRNA expression revealed that *C. krusei* infected mice presented an elevated expression of many pro-inflammatory markers, including some as Tbx21, IFN-γ and TNF-α that highly suggested a local Th1 polarization. Additionally, mRNA of pattern recognition receptors (PRRs) as dectin-1 and toll-like receptor (TLR)-2, present in innate immune cells, was increased in immunosuppressed mice, and especially in mice infected with *C. krusei*. Dectin-1 is a PRR that binds β-glucans and collaborates with TLR-2 receptor for inflammatory activation of phagocytes against *Candida* spp., including macrophages (Gantner et al., 2005; Xu et al., 2015), that are predominantly activated by Th1 subset.

To further understand the host-parasite interaction in CNS, we tested NO production and yeast-to-hypha or pseudohypha transition in microglial cells lineage infected with *Candida* species. Microglia is the resident phagocyte of the CNS and this cell type was chosen because it is the main cell involved in the control of fungal colonization in the CNS (Koutsouras et al., 2017). The results indicated that *C. krusei* was very distinct from the two other non-*albicans Candida* species; it induced much higher NO levels and was the only one able to switch from yeast-to-pseudohypha.

Innate immune cells as macrophages and microglia produce reactive oxygen and nitrogen (ROS and RNS) species, including high levels of NO, against pathogens. However, pathogens have evolved many strategies to escape from NO effects. A wide variety of microbes including bacteria and fungi, especially *C. albicans*, can detoxify NO using flavohemoglobins detoxification enzymes, that confer resistance to NO microbicidal effect (Hromatka et al., 2005). In our study, while very low NO levels were found in *C. albicans* infected microglia cells, *C. krusei* infection triggered a significantly higher NO production. This is interesting because NO is a key regulator of T-bet expression and was directly associated to Th1 subset expansion and Th2 subset suppression (Pahan and Mondal, 2012). In addition, *C. krusei* and *C. parapsilosis* are being consistently the most sensitive species to NO (McElhaney-Feser et al., 1998).

Our findings reveal that *C. krusei* uses different strategies to try to escape from the immune response. This species is able, for example, to switch from yeast-to-pseudohypha inside microglial cells while *C. glabrata* and *C. parapsilosis* remain in the yeast form inside these cells. Moreover, it is already described that *C. krusei* induces vomocytosis to avoid destruction by macrophages (García-Rodas et al., 2011). This process consists of fungal expulsion from the macrophage without lysing the host cells (Gilbert et al., 2015). A similar process was reported in *C. albicans* (Bain et al., 2012) and corroborates our *in vitro* data. In summary, we hypothesize that *C. krusei* early induction of NO could contribute to Th1 differentiation. However, this species would be able to escape from macrophage’s microbicidal activity by vomocytocis.

Altogether these results indicate that *C. glabrata*, *C. krusei* and *C. parapsilosis*, similarly to *C. albicans*, are also able to disseminate to the CNS and promote local inflammation. In addition, the findings clearly show that corticotherapy increases their respective fungal loads at both, the brain and the spinal cord, being *C. krusei* the species that triggers the highest local accumulation of Th1 cells. It is possible that the neuroinflammation triggered by this type of Th1-inducing *Candida* is even more deleterious to CNS pathologies originally associated to Th1 immunopathogenesis. Considering also that CNS samples are difficult to obtain from patients, we believe that experimental models can be widely explored to investigate the effect of these fungi infections in many CNS disorders such as multiple sclerosis, Parkinson’s and Alzheimer’s diseases.

## Ethics Statement

The animals were manipulated in accordance with the Brazilian legislation that is regulated by the National Council for the Control of Animal Experimentation (CONCEA) and by the Ethical Principles in Animal Research formulated by the Brazilian Society of Science in Laboratory Animals. The whole experimental protocol was also approved by the Medical School Ethics Committee on Use of Animals (CEUA – protocol number 1206/2017), UNESP, Botucatu, SP, Brazil.

## Author Contributions

MS, AS, CK, and TF-S conceived and designed the experiments. MS, LM, LO, LI, and TF-S performed the experiments and contributed to immunological and histopathological analysis tools. HG, EB, and TF-S contributed to biofilm and bioinformatics analysis tools. MS, AS, and TF-S analyzed the data and wrote the manuscript. All authors approved the final version to be published.

## Conflict of Interest Statement

The authors declare that the research was conducted in the absence of any commercial or financial relationships that could be construed as a potential conflict of interest. The reviewer RR declared a shared affiliation, with no collaboration, with the authors HG and EB, to the handling Editor at the time of review.

## Footnotes

1 https://blast.ncbi.nlm.nih.gov/Blast.cgi
